# Plant-Derived Antimicrobial Peptides: Novel Preservatives for the Food Industry

**DOI:** 10.3390/foods11162415

**Published:** 2022-08-11

**Authors:** Piyush Baindara, Santi M. Mandal

**Affiliations:** 1 Departments of Molecular Microbiology & Immunology, School of Medicine, University of Missouri, Columbia, MO 65211, USA; 2 Central Research Facility, Indian Institute of Technology Kharagpur, Kharagpur 721302, West Bengal, India

**Keywords:** plant antimicrobial peptides, food preservatives, food spoilage, foodborne pathogens, peptide micelles

## Abstract

Food spoilage is a widespread issue brought on by the undesired growth of microbes in food products. Thousands of tons of usable food or food products are wasted every day due to rotting in different parts of the world. Several food preservation techniques are employed to prevent food from rotting, including the use of natural or manufactured chemicals or substances; however, the issue persists. One strategy for halting food deterioration is the use of plant-derived antimicrobial peptides (AMPs), which have been investigated for possible bioactivities against a range of human, plant, and food pathogens. The food industry may be able to benefit from the development of synthetic AMPs, produced from plants that have higher bioactivity, better stability, and decreased cytotoxicity as a means of food preservation. In order to exploit plant-derived AMPs in various food preservation techniques, in this review, we also outline the difficulties in developing AMPs for use as commercial food preservatives. Nevertheless, as technology advances, it will soon be possible to fully explore the promise of plant-derived AMPs as food preservatives.

## 1. Introduction 

Food waste caused by microorganisms such as bacteria, fungi, and yeast is on the rise globally in the modern era. The Food and Agriculture Organization (FAO) defines food loss and waste as a “decrease in quality and quantity of food along with the food supply chain.” One-third of the worldwide food supply generated for human consumption, or 1.3 billion tonnes per year, is wasted, according to the World Food Program (WFP) (https://www.wfp.org/stories/5-facts-about-food-waste-and-hunger (accessed on 30 July 2022)). According to a study, food spoiling caused by spoilage bacteria results in a loss of 25% of the food produced globally [1]. Food spoilage is a complex process in which microorganisms play a major role. The process of food spoilage includes the growth and reproduction of microorganisms along with physical, chemical, and enzymatic reactions with food via different processes including, lipid oxidation, proteolysis, starch aging, and postharvest respiration in vegetables, where enzymes such as polyphenol oxidase, pectinase, and lipases are playing important roles [2,3]. The growth of microorganisms during food spoilage depends on the type of substrate available in the food along with water, sugar, salt, oxygen, and nutrient content [4].

Food loss and wastage are still issues despite the adoption of food preservatives and preservation techniques such as cold storage, improved packaging, ionization, and chemical and natural food additives [5]. Chemical food preservatives are extensively used worldwide because of their low cost and straightforward manufacturing procedure. Food preservatives play a crucial part in the fight against food degradation. However, the inappropriate use or prolonged consumption of chemical preservatives including sodium benzoate, sodium nitrate, sodium nitrite, and benzoic acid has been linked to significant health problems [6,7]. Facts about the adverse effects of using chemical preservatives in food result in the development of concern in modern consumer behavior and an increased interest in natural biological preservatives. Essential oils, organic acids, chitosan, plant extracts, and bioactive AMPs from animals, bacteria, and plants are the major interesting alternatives to chemical food preservatives. Research on natural food preservatives has already shown that they may be more effective than chemical food preservatives [8,9]. It is interesting to note that some naturally occurring AMPs and their synthetic analogs have already been employed as food preservatives in the food industry [10].

AMPs are proteinaceous compounds produced by almost every form of life, including plants, animals, and microorganisms, with the biological function of self-protection against pathogens [11,12,13]. Self-protection is essential for survival in plants, as they are exposed to various pathogens in different seasons throughout the year [14,15]. Due to the necessity of survival via self-protection against microorganisms, plants are considered considerable sources of AMPs [16]. Several plant AMPs have been reported from underground as well as all aerial parts, including, flowers, fruits, leaves, bark, and seeds, with potential and diverse bioactivities, especially antibacterial, anti-yeast, and antifungal activities. Besides their potential bioactivities, plant AMPs have the benefits of safety, good curative effects, and selectivity; therefore, researchers around the world attempted to explore their use in the food industry and other biotechnological applications [17]. Here, we outlined the potential of plant AMPs for use in the food sector to enhance food preservation and shelf life. Information was collated on plant AMPs that have demonstrated potential antibacterial activity against several foodborne diseases, and some of these compounds have already been used in the food business. In addition, we explored the advantages and disadvantages of using plant AMPs as food preservatives in the food sector. The overall conclusion of this analysis pointed to plant AMPs’ promise in a variety of food preservation applications for the present and future food sector.

## 2. Different Classes of Plant-Derived AMPs

### 2.1. Defensins

**Figure 1:**
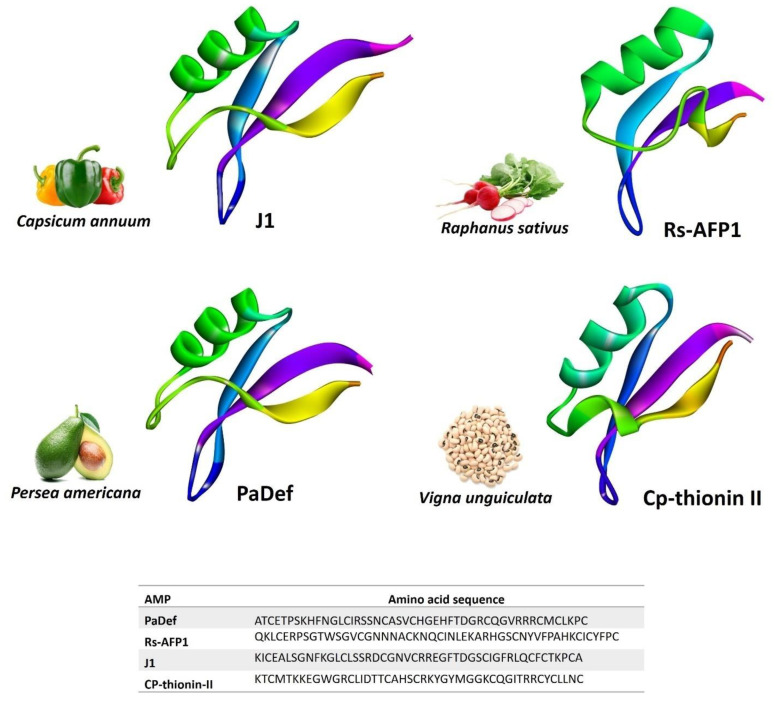
Selected plant defensins that showed potential antimicrobial activities for food preservation: J1 (predicted in this study), Rs-AFP1 (PDB ID: 1AYJ), PaDef (predicted in this study), and Cp-thionin II (predicted in this study). Predicted structures are modeled via homology modeling using the Swiss model (https://swissmodel.expasy.org accessed on 30 July 2022).

Defensins are universal in the kingdom of plants and are present in all life forms. Defensins are brief peptides that range in size from less than 45 amino acids in length to a molecular weight of about 5 kDa [18,19,20]. Plant defensins are very basic, and the formation of disulfide linkages has been linked to 8–10 cysteine residues [21]. Plant defensins are kept together by four disulfide linkages and have a distinct, three-dimensional structure. According to their 3D structure, plant defensins have a parallel, triple-stranded β-sheet and α-helix [22,23]. Due to their antibacterial and antifungal capabilities, defensins are produced by plants during their reproductive, storage, and stress responses and serve to protect them [24,25] (Figure 1). 

### 2.2. 2S Albumins

**Figure 2:**
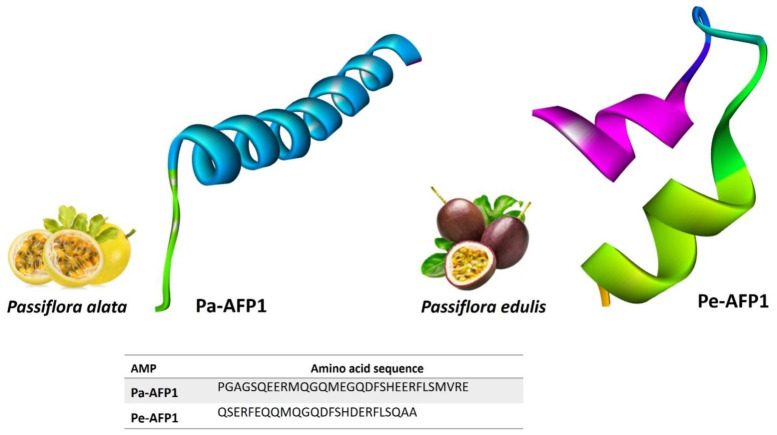
Selected 2S albumin proteins that have potential antimicrobial activities for food preservation: Pa-AFP1 (predicted in this study) and Pe-AFP1 (predicted in this study). Predicted structures are modeled via homology modeling using the Swiss model (https://swissmodel.expasy.org accessed on 30 July 2022).

The term 2S albumin comes from the sedimentation coefficient of the protein. These are storage proteins that are necessary for plant development and growth. Notably, 2S albumins are made up of a large 18–20 kDa precursor peptide that is post-translationally modified through proteolytic cleavage to yield small active peptide fragments [26]. Finally, the processed AMP accumulates in storage vacuoles inside seeds, leaves, and vegetative tissues such as tubercles and has a low molecular weight that ranges from 4 to 9 kDa. Importantly, 2S albumins have been discovered to exhibit antibacterial and antifungal properties [27] (Figure 2).

### 2.3. Glycine-Rich Proteins (GRPs)

**Figure 3:**
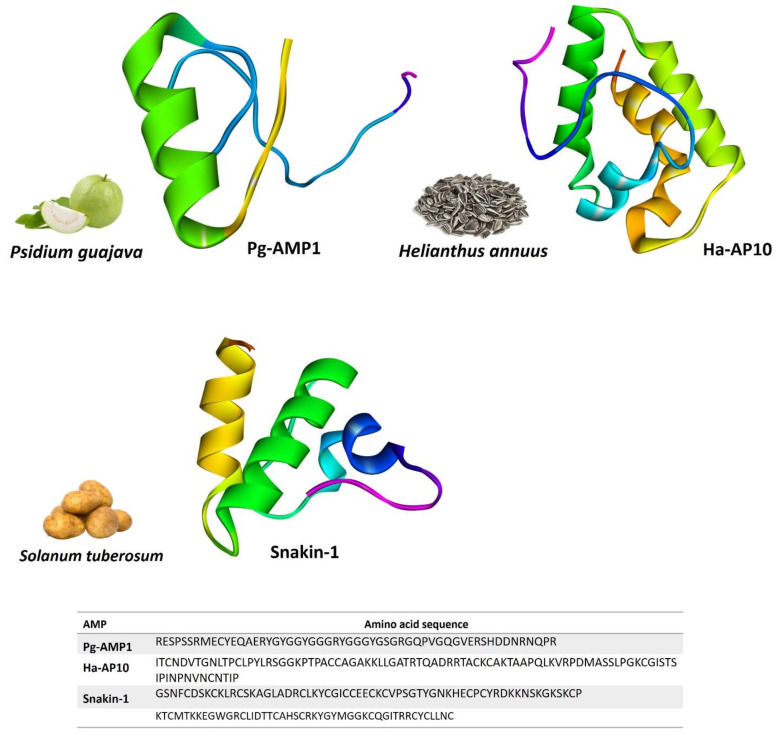
Selected glycine-rich, lipid transfer, and snakin proteins that showed potential antimicrobial activities for food preservation: Pg-AMP1 (predicted in this study), Ha-AP10 (predicted in this study), and snakin-1 (PDB ID: 5E5Q). Predicted structures are modeled via homology modeling using the Swiss model (https://swissmodel.expasy.org accessed on 30 July 2022).

GRPs are storage proteins found in the xylem, hypocotyls, stems, and petioles of plants [28]. They are frequently characterized by several glycine-containing motifs and an overall 70% of glycine percentage in the protein. Plants use GRPs to defend themselves against abiotic and biotic stress through their antifungal and antibacterial properties [29] (Figure 3). 

### 2.4. Lipid Transfer Proteins (LTPs)

LTPs are lipid carriers that bind monomeric lipid units in a hydrophobic pocket and play essential roles in lipid transfer across donor and acceptor membranes [30]. They are small proteins with a molecular weight of less than 10 kDa. Like defensins, LTPs are cysteine-rich and have four to five α-helices with four disulfide linkages. Several disulfide connections give LTPs their high degree of stability and resistance to heat denaturation [31,32]. Due to their antibacterial qualities, LTPs are recognized to be essential for plant survival and play significant roles in plant breeding [33] (Figure 3). 

### 2.5. Snakins

Snakins are low-molecular-weight peptides with 12 cysteine residues that are involved in disulfide bond formation and are highly conserved plant AMPs [34]. They are known to have potent antibacterial and antifungal properties against a variety of plant diseases, as well as being involved in plant development and growth [35] (Figure 3).

### 2.6. Thionins

Thionins are small peptides consisting of 45–48 amino acids with 3–4 disulfide bonds and about 5 kDa of molecular weight. Thionins are usually found in higher plants and are reported to have potent antibacterial and antifungal activities [36].

### 2.7. Cyclotides

Cyclotides are small cysteine-rich AMPs isolated from plants. They have three characteristic disulfide bonds and usually contain 28–37 amino acids. They are known as cyclotides because of their head-to-tail cyclized peptide bone [37]. Cyclotides have a variety of biological effects, including antibacterial, antifungal, insecticidal, and anticancer effects [38]. 

### 2.8. Napins

**Figure 4:**
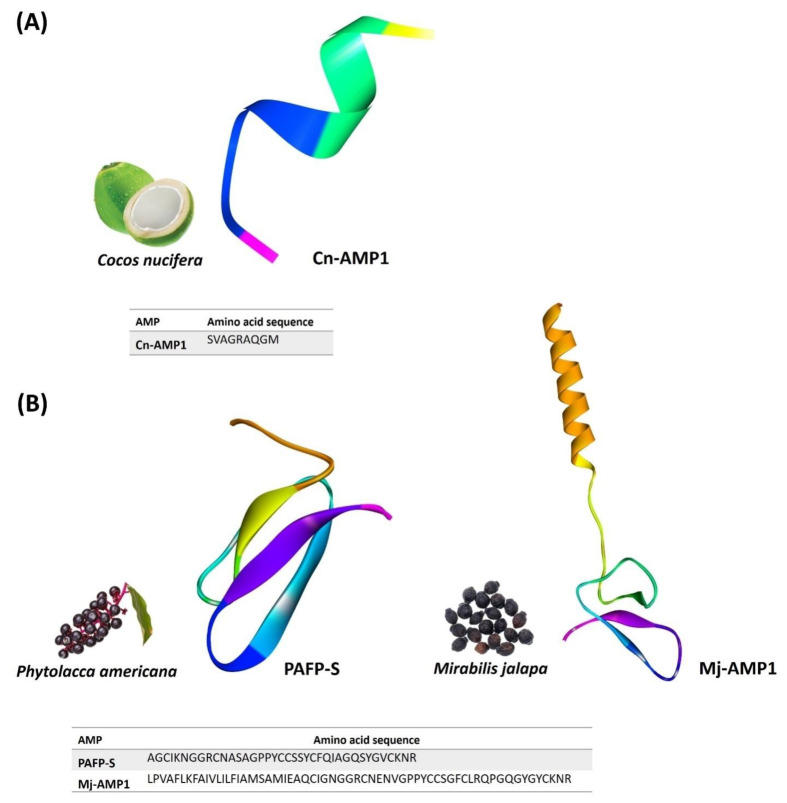
Selected napin (**A**) and knottin-like (**B**) plant AMPs that have potential antimicrobial activities for food preservation: Cn-AMP1 (PDB ID: 2N0V), PAFS-S (PDB ID: 1DKC), and Mj-AMP1 (predicted in this study). Predicted structures are modeled via homology modeling using the Swiss model (https://swissmodel.expasy.org accessed on 30 July 2022).

Napins are low-molecular-weight plant AMPs, with a molecular weight of less than 15 kDa, while two polypeptide chains are linked by disulfide bonds. Like defensins and other plant AMPs, napins are also cysteine-rich peptides [39]. Napins are seed storage proteins with high water solubility [40]. They are reported to have antibacterial, antifungal, and trypsin-inhibiting properties in addition to their role in plant growth and development [41] (Figure 4A).

## 3. Antagonistic Effects of Plant-Derived AMPs against Foodborne Pathogens

Plant AMPs could be a potential alternative for biopreservation applications in the food industry. They can be used for the development of high-yielding genetically modified crops with enhanced resistance against plant and foodborne pathogens. Plant AMPs have already been suggested as potential drug sources for human infectious diseases caused by bacteria, viruses, and parasites, and for the treatment of different types of cancers [42,43,44]. Although the use of plant AMPs in a clinical setting requires further in-depth experimental studies, they are suitable potential options for use in the food industry as food preservatives against foodborne pathogens. In the natural environment, plants experience different weather and drought conditions, and therefore, their immune system is developed to efficiently fight against various pathogens, where AMPs play essential roles in their protection [45]. 

It is interesting to note that using plants and the products in which they are utilized, such as natural medicines and cosmetics, has long been linked to improving human health. Additionally, a number of the pharmaceuticals we use today are derived from plants [46]. Furthermore, the numerous antibacterial qualities of AMPs generated from plants suggest their potential use in agricultural production [47]. Additionally, the promising antifungal and antibacterial properties of AMPs generated from various plant components have the potential to be developed for biotechnological uses in the food business, such as food preservatives [48,49]. 

To date, various plant-derived AMPs have been isolated and characterized with enormous structural and functional diversity (Table 1). PaDef was discovered and extracted from a cDNA library obtained from Mexican avocado fruit. It is a peptide that resembles a defensin. PaDef can be utilized to treat foodborne infections because it has antibacterial action against Escherichia coli and Staphylococcus aureus [50]. Another defensin, J1, was discovered in a cDNA library of bell pepper fruit and demonstrated potent antifungal activity against the *Colletotrichum gloeosporioides*-induced anthracnose disease of transgenic bell pepper [51,52]. Similar to this, Rs-AFP1 and Rs-AFP2, two defensins isolated from radish seeds, have potent antifungal action [53].

Further, these two defensins were chemically synthesized and reported to have anti-yeast activity against food spoilage yeast *Zygosaccharomyces bailii* in different beverages [54]. In another study, four closely related cysteine-rich peptides were isolated and characterized from the seeds of *Impatiens balsamina* (Balsam), showing antifungal and antibacterial activities [55]. Interestingly, these cysteine-rich peptides did not show any cytotoxicity against human cells while exhibiting strong activity against enteric foodborne pathogens including *S. aureus*, *E. coli*, *Salmonella enterica*, *Pseudomonas aeruginosa*, and *Bacillus cereus* [56]. Plant-derived AMPs can also be used for the protection of stored grains in warehouses, as reported in a study where a defensin, Cp-thionin-II, isolated from cowpea seeds, was confirmed to protect stored wheat grains from fungal spoilage caused by *Fusarium culmorum* [57]. MsDef1 and MtDef4 are plant defensins isolated from *Medicago sativa*, and *M. truncatula* showed inhibitory activity against *F. graminearum*, a fungal plant pathogen that caused fusarium head blight of wheat and barley [58]. 

Another class of AMPs identified in plants is 2S albumin proteins. Pa-AFP-1 is isolated from passion fruit and has been found to efficiently inhibit the development of filamentous fungi, *C. gloeosporioides*, *Trichoderma harzianum*, *F. oxysporum*, and *Aspergillus fumigatus* [27,59]. CW-1 is another 2S albumin protein, isolated and characterized from *Malwa perviflora* (Cheeseweed), that is reported to have antifungal activity against *F. graminearum* [61]. 

Pg-AMP1 is a glycine-rich peptide that is isolated from guava seeds and has been shown to have potent antibacterial properties against Klebsiella species and Proteus species [62]. A class of plant AMPs called lipid transfer proteins is effective against bacteria, yeast, and fungi. The lipid transfer protein Ca-LTP1, which is isolated from chili pepper seeds, has high antifungal action against *C. lindemunthianum* and *C. tropicalis* and may be employed as a food preservative [63]. 

Another lipid transfer protein identified from sunflower seeds is called Ha-AP10. Ha-AP10 demonstrates strong inhibitory action against the germination of spores of pathogenic fungal pathogens *F. solani*, indicating its potential use in the food business [64]. Next, a mung bean nsLTP, isolated from mung bean sprouts, has been reported for its potential antifungal activities against various fungi, including *F. solani*, *F. oxysporum*, *Pythium aphanidermatum*, and *Sclerotium rolfsii*, as well as its antibacterial activity against *S. aureus*.

Snakin-Z is identified and isolated from Jujube fruits and exhibits potential antibacterial and antifungal activities against *S. aureus* and *Phomopsis azadirachtae*, respectively. Interestingly, snakin-Z does not exhibit any cytotoxicity against RBCs and is suggested as a potential plant AMP for therapeutic or food preservation applications [67]. MsSN1 is a snakin-1, isolated and characterized from *M. sativa*, that exhibits antibacterial and antifungal activity against multiple foodborne pathogens [70]. 

Next, CaThi is a thionin-like peptide characterized and isolated from chili. CaThi is reported to have antimicrobial, anti-yeast, and antifungal properties against various pathogenic bacteria, including *S. cerevisiae*, *Candida albicans*, *C. tropicalis,* and *F. solani* [71,89]. Another plant thionin is isolated from the wheat endosperm material that showed antibacterial activities against *Corynebacterium michiganense* and *Xanthomonas campestris*, which are known plant pathogens of tomatoes and peppers [72,90]. Another study reported a potent antifungal thionin named thionin 2.4, secreted from *Arabidopsis thaliana*, which showed antifungal properties against *F. graminearum*, a serious crop fungal pathogen [73]. TuAMP1 and TuAMP2 are thionin-like peptides that are isolated and characterized from the bulbs of *Tulipa gesneriana* (Tulip) and exhibit diverse antifungal activity against *Agrobacterium rhizogenes*, *A. radiobacter*, *Clavibacter michiganensis*, *Curtobacterium flaccumfaciens*, *F. oxysporum*, and *Geotrichum candidum* [74].

Cycloviolacin O2 and Cycloviolacin O8 are cyclotides isolated and characterized from *Viola odorata*, which showed potential antibacterial activity against various pathogenic bacteria [75,77]. Additionally, parigidin-br1, a cyclotide isolated from *Palicourea rigida*, is reported to have potent insecticidal activity against neonate larvae of Lepidoptera (*Diatraea saccharalis*), a sugarcane insect [91]. This suggests that plant-derived AMPs can also be developed as biopesticides for direct applications in food crops.

**Figure 5:**
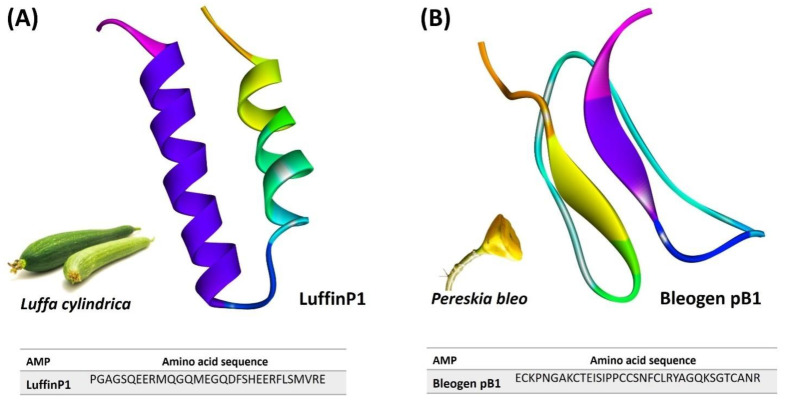
Selected α-Hairpin-like peptide (**A**) and hevein-like peptides (**B**) that have potential antimicrobial activities for food preservation: LuffnP1 (PDB ID: 2L37) and Bleogen (PDB ID: 5XBD).

An α-hairpin-like peptide, LuffinP1, isolated and characterized from the seed of *Luffa cylindrica* (Sponge gourd), showed potential protein translational inhibitory activity [78]. Bleogen pB1 is a hevein-like peptide isolated from Cactus fruits and has been reported to have potent inhibitory activity against *C. albicans* and *C. tropicalis* [79] (Figure 5A and 5B. Next, two hevein-like peptides, EAFP1 and EAP1, isolated and characterized from the bark of *Eucommia ulmoides*, exhibit broad inhibitory activity against eight pathogenic fungi from cotton, wheat, potato, tomato, and tobacco [80]. Another hevein-like peptide, Ee-CBP, was purified and characterized from the bark of *Euonymus europaeus* and showed broad-spectrum antifungal activity against various plant pathogenic fungi [81]. SmAMP3 is a novel hevein-like peptide that is isolated from leaves of *Stellaria media* and has been reported to have potent inhibitory activity against plant pathogenic fungi [82].

Napins are another class of plant AMPs that show promising results to be used as food preservatives. Em2-F18 is a napin-like peptide isolated and characterized from the seed of Jambu fruits, confirmed to have potential antibacterial activities against foodborne pathogens, such as *S. aureus* and *S. enteritidis* [83]. Another napin, Tn-AFP1, was identified from the Water chestnut and found to have antifungal activity against *F. oxysporum*, *Mycosphaerella arachidicola,* and *Physalospora piricola* through its inhibition of fungal mycelial growth [84]. Mandal et al. identified and characterized two other napin-like peptides, Cn-AMP1 and Cn-AMP2, from green coconut, which were found to exhibit potent antifungal activity against *C. tropicalis* [85].

Knottin-type peptides are another class of plant-derived AMPs that show strong antifungal properties against foodborne pathogens. PAFP-S is a knottin-like peptide isolated from the seeds of *Phytolacca Americana* and exhibits antifungal activities against *F. oxysporum* and *Pyricularia oryzae*, which are common crop pathogens of legumes, rice, and barley [86]. Mj-AMP1 and Mj-AMP2 are other knottins isolated from the seeds of *Mirabilis jalapa* and exhibit potential antifungal activity against major pathogenic fungal species, including *F. oxysporum*, which causes significant loss to crops [87] (Figure 4B) (Table 1). Many unclassified plant AMPs, in addition to the known classes of plant AMPs, have the potential to be used in the food sector. A plant AMP called Cn-AMP3 was discovered in coconut water and demonstrated promising antibacterial properties against various bacterial species [88]. The aforementioned examples collectively show the potential of plant-derived AMPs against several plant diseases and foodborne pathogens, which can be further developed as food preservatives in the future food industry to prevent food spoiling or loss.

## 4. Applications of Plant-Derived AMPs in the Food Industry

Plant-derived AMPs have been proven to possess strong antibacterial capabilities against bacteria, yeast, and fungi, pointing to the possibility of using them in the future to create food preservatives for the food industry. However, natural plant AMPs have several disadvantages that prevent their usage in culinary applications. The main drawbacks of natural plant AMPs include poor chemical stability, astringent flavor, short-term effectiveness, and cytotoxicity. Despite all the negative aspects, plant AMPs can be altered or enhanced for use as food preservatives through chemical synthesis and the addition of delivery techniques such as encapsulation, nanoparticles, or edible packaging [48,92]. With recent developments in technology and scientific advancement, some of the natural plant AMPs have been modified and used in the food industry with improved efficacy (Table 2). In a recent study, glycinin basic polypeptides (GBPs) were isolated from soybean and found to have strong antifungal properties against *A. niger* and *Penicillium* sp. by inhibiting fungal mycelial growth, spore germination, and plasma membrane disruption via inhibition of ergosterol synthesis. By improving the sensory qualities of fresh, wet noodles, this GBP intriguingly demonstrated possible food preservation characteristics and has been proposed as a potential tool for extending the shelf life of starchy foods [93]. Ning et al. demonstrated the effects of soybean GPB’s preservation properties on the *Scomberomorus niphonius surimi* (Japanese Spanish Mackerel). In a 24-day experiment, it was proven that GBP enhanced texture and prevented microbiological growth, extending the overall shelf life of surimi when stored [94].

**Figure 6:**
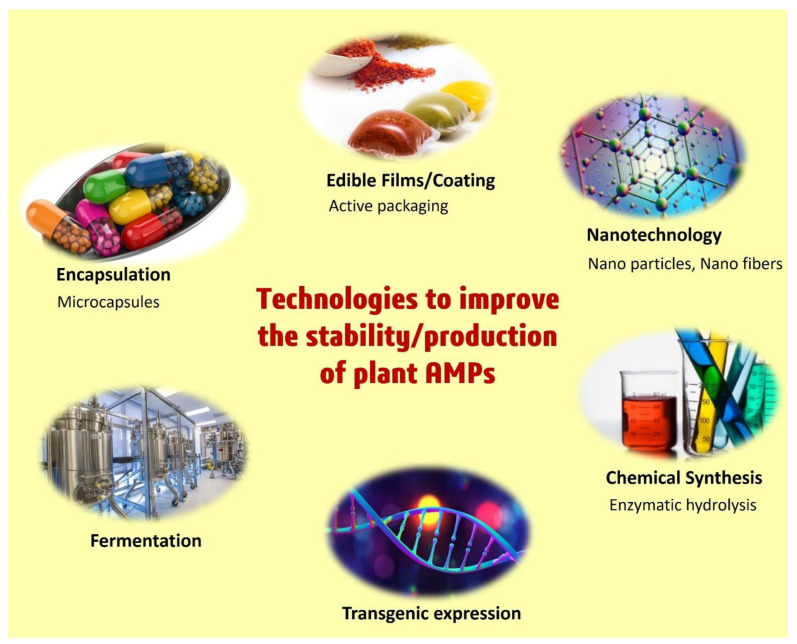
Different scientific technologies for the stability and production improvement of raw, processed, and liquid food, using plant AMPs.

Another study aiming at the evaluation of food preservative capacities of legumes (pea, lentil, and fava bean flours) reported the potent antifungal activity of this native flour mixture against multiple species of *Aspergillus* and *Penicillium*. Further, purification confirmed the presence of nine native peptides in the legume flour mixture. A purified blend of these nine peptide mixtures was used to make bread under pilot plant conditions, which showed a longer shelf-life in comparison to the control bread [95]. In another study, the same group found the antifungal activity of pea hydrolysate against *P. roqueforti* and found the mixture of active components as a blend of pea defensins, lipid transfer proteins, and other peptides. They synthesized the identified peptides and found that this mixture is efficiently able to enhance the shelf-life of bread up to the storage period of 21 days [100]. Low-molecular-weight peptides that have the ability to extend the shelf life of starchy foods and exhibit antifungal action were also discovered to be produced during the fermentation of plant products (Figure 6). Palm kernel cake is fermented via solid-state Lacto-fermentation by using *Lactobacillus casei*. The generated fermentation product was identified as a mixture of peptides produced by Palm kernels and bacteria. The peptide mixture was reported to have antifungal activity against *Aspergillus* sp., *Fusarium* sp., and *Penicillium* sp. while also found to enhance the shelf-life of whole wheat bread slices for a storage period of 10 days [96]. 

Another method for producing plant-derived AMPs on a large scale is recombinant expression. Huang et. al. confirmed the recombinant expression and production of Ac-AMP2, a plant AMPs, originally produced by *Amaranthus caudatus* (an annual flowering plant), and MiAMP1, a highly basic protein from the nut kernel of *Macadamia integrifolia*. The recombinant strains of *Pichia pastoris* (GS115/Ac-AMP2 and GS115/MiAMP1) expressing these peptides showed potential post-harvest food preservation properties in pears infected with the fungal pathogen *P. expansum* [97].

Another method for preserving food during lengthy storage is to use food preservation films coated with AMPs. By using several encapsulation techniques such as liposomes, emulsions, biopolymer particles, nanofibers, and nanofilms, plant AMPs can be employed in active food packaging [10] (Figure 6). A recent study created and tested double-layered furcellaran/gelatin hydrolysate films containing the Ala-Tyr peptide for the preservation of frozen fish. No bacterial growth or oxidation was seen throughout the lengthy four-month storage period at −18 °C [98].

Another recent study showed the food preservation potential of plant AMPs in minced meat. A peptide isolated and characterized from *Momordica charantia* L. (Bitter melon) seeds exhibited antibacterial activity against *E. coli*, *S. aureus*, *S. typhi*, and *P. aeruginosa*. Further, minced meat samples treated with this peptide showed significant bacterial count reduction over time, suggesting the food preservation potential of this peptide [99]. *Zygosaccharomyces* species are known for causing the spoilage of high sugar-containing food products such as fruit juice and carbonated drinks. Shwaiki et al. reported that snakin-1, isolated from potato tubers, showed anti-yeast activity against *Zygosaccharomyces* sp. through membrane permeabilization. Sankin-1 was found to protect the spoilage of beverages (Fanta orange, cranberry juice, and apple juice), inoculated with *Zygosaccharomyces* sp., by inhibiting the yeast growth in peptide-treated samples [69] (Table 2). 

**Figure 7:**
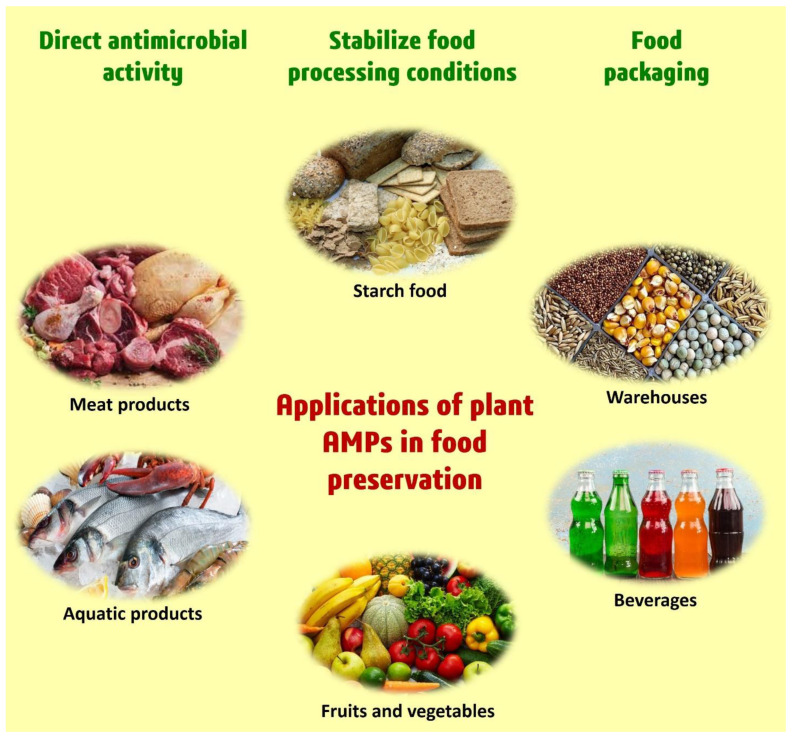
Different applications of plant AMPs in raw, processed, and liquid food.

Finally, as an alternative to conventional chemical food preservatives, plant AMPs with promising antibacterial capabilities may be taken into consideration. Even though few studies have been conducted thus far on the potential of plant AMPs for the food industry, their potential and promising properties against foodborne pathogens, combined with the enormous diversity already present, point to them as potential food preservatives in the near future (Figure 7). 

## 5. Transgenic Expression of Plant-Derived AMPs to Fight against Plant Pathogens

Peptides are made up of amino acids, and therefore, their cloning, expression, and genetic manipulation are easy. Transgenic expression of potential plant AMPs in other edible plants or crops is another strategy to fight against foodborne pathogens. It has been reported that the transgenic expression of the *C. annuum* (Chilli) defensin gene in tomato plants leads to resistance against plant pathogenic fungi, *Fusarium* sp. and *Phytophthora infestans* [101]. In another, study, when *Medicago sativa* defensin gene *MsDef1* was integrated and expressed in *Fusarium*-susceptible *Lycobersicum esculentum* Mill cultivar CastleRock (tomato), transgenic lines were found to have resistance against *Fusarium* wilt [102]. The floral defensins of petunia (*PhDef1* and *PhDef2*) were also found to provide efficient resistance against *F. oxysporum* when overexpressed in banana plants [103]. The overexpression of another defensin gene (Sm-AMP-D1) from *Stellaria media* (common chickweed) has also been reported to protect against *F. oxysporum* in banana plants [104]. *Phytophthora* sp. is a major fungal plant pathogen and causes huge economic loss worldwide. The ectopic expression of a defensin gene (DmAMP1) from *Dahlia merckii* in papaya plants exhibits resistance against *P. palmivora* infection and results in reduced growth of fungal hyphae at the infection site [105]. Further, the overexpression of defensin genes with other genes has been reported for the protection against fungal pathogens. Chen et al. reported that when tobacco β-1,3-glucanase gene (GLU), alfalfa defensin gene alfAFP, and their bivalent gene GLU-AFP transformed in tomato plants, transgenic tomato lines having GLU-AFP cassette showed the highest resistance against *Ralstonia solanacearum* [106]. A similar strategy was applied to tomato plants using the rice chitinase gene (CHI) with the alfalfa defensin gene alfAFP, which resulted in highly resistant transgenic tomato lines (CHI-AFP cassette) against *Borytiscyneria* [107]. These studies suggest that plant-derived AMPs genes such as defensin genes can provide resistance against phytopathogenic fungi, and their transgenic expression can be used for the development of highly resistant and high-yielding varieties of fruits, vegetables, and crops (Figure 6).

## 6. AMP Micelles and Foodborne Pathogens in the Near Future

Plant-derived AMPs have been shown to have promising antibacterial capabilities against a variety of foodborne pathogens, suggesting that they might be used as food preservatives in the food industry (Table 1 and Table 2). In reality, it has always been challenging to deliver AMPs to the targeted foodborne pathogens in food in a precise and secure manner. The controlled release of AMPs over time to achieve the best food preservation effects is another significant issue. As it reduces the possibility of dangerously high concentrations and enables AMPs to exit for a longer period, controlled and targeted AMP release is essential in food preservation [108].

A recently proposed method for peptide delivery in medical applications uses micelles that include AMPs. A potential method to increase the efficiency and usefulness of AMPs as food preservatives for a longer period is the development of plant-derived AMP micelles.

Furthermore, AMP micelles are simple to make, and the size and charge of the micelles may be readily regulated during creation by adjusting the hydrophilic and lyophilic balance. AMPs quickly self-associate with micelles, preventing them from aggregating. Finally, because self-association has no chemical effect on AMPs, it does not denature or limit their bioavailability or bioactivity [109]. AMP micelles also have the advantage of being easy to prepare on a large scale, which makes them appealing for a smooth changeover in the food industry [110]. The Food and Drug Administration (FDA) has authorized PEG-PLA micelles, which have been thoroughly examined for biodegradation and biocompatibility [111]. The promising drug delivery capabilities of AMP micelles present them as a viable method for using plant AMPs as food preservatives against foodborne pathogens in the future food industry. In addition, AMP micelles could be used in various food packaging materials for the elimination of foodborne pathogens [10]. Furthermore, employing different AMP micelles containing different AMPs can target different foodborne pathogens simultaneously present in the food.

## 7. Challenges for the Development of Plant-Derived AMPs as Food Preservatives

Thus far, several AMPs have been found with the potential to be employed as food preservatives or in other biotechnological applications; however, only a handful have been authorized for clinical use or human ingestion, and the majority are still in clinical testing [112]. As natural AMPs cannot be directly employed for a variety of reasons (Table 3), virtual screening approaches and strategies to produce synthetic AMPs based on natural AMPs are needed for AMPs to have a bright future. AMPs are composed of amino acids, which gives allows them to be bioengineered to improve antimicrobial capabilities. This ability to change or improve AMPs makes them a potential and prospective choice for the food industry and other biotechnological applications. AMPs are often synthesized in harsh settings and have high endurance for a wide range of temperatures, pH levels, and salt concentrations, making them viable contenders for use in the food sector [12].

For the industrial development of AMPs, in-depth knowledge or establishment of essential peptide synthesis techniques is required. Additionally, numerous AMPs have already been discovered, and there are many opportunities for additional AMPs to be discovered, which might serve as the foundation for a better synthetic version with the robust antimicrobial capabilities that are needed. Another good element is today’s customer perspective in the food industry, which has boosted the need or demand for natural products or preservatives over the use of artificial and chemical preservatives. In comparison to the cheap market cost of conventional food preservatives such as sodium benzoate, the cost of synthetic AMPs is too exorbitant to bring them into industrial-scale manufacturing, despite their various benefits and potential antimicrobial capabilities. In the current situation, the expense of the chemical synthesis of AMPs is a major stumbling block to their use in the food industry or other biotechnological applications. Over time, specific technological developments may boost the prospects of low-cost AMP production and their widespread availability as food preservatives or other biotechnological applications (Figure 6). The current examination of the features and limitations of AMPs for commercial development adds further insight to their future development as food preservatives in the food industry.

## 8. Conclusions

Food waste and food deterioration are both worldwide problems that need to be currently addressed in this period of economic crisis and population growth. Although traditional methods of food preservation are still useful and contribute to the decrease in food waste, foodborne germs continue to be an issue because they cause food to spoil. The current analysis emphasized the potential use and development of plant-derived AMPs as food preservation agents for the food sector to reduce food waste (Figure 7). Plant-derived AMPs were recommended as viable candidates for the creation of future food preservatives due to their diverse structural features and potential antibacterial, anti-yeast, and antifungal actions. 

For example, starch-based foods, warehousing items, meat, aquatic products, beverages, fruits, and vegetables can all benefit from having strong inhibitory actions against foodborne pathogens (Figure 7). Furthermore, the elimination of plant pathogens through the transgenic expression of plant AMPs in fruits, vegetables, and crops has the potential to boost output. Additionally, the use and advancement of AMP-based micelles in the fight against foodborne pathogens in the near future point to them as possible contenders for future food preservative development (Figure 6).

Although there are numerous obstacles in the way of plant AMPs becoming widely used as food preservatives, with the rapid growth of science, it is now possible to fully explore the potential of plant-derived AMPs for the food business of the future. Overall, this research demonstrated that plant AMPs have the potential to have bioactivities against foodborne infections and that more effective synthetic counterparts with more stability and bioactivities may be created as industrial food preservatives. To ascertain the long-term cytotoxicity and harmful effects of plant-derived AMPs on human health, more analysis and clinical trials are necessary.

## Figures and Tables

**Table 1:** Different classes of plant-derived AMPs, sources, and antimicrobial activity.

Class	Name	Source	Antimicrobial Activity	References
Defensins	PaDef	Avocado (*P. americana*)	*E. coli* *S. aureus*	[50]
	J1	Fruit peppers (*C. annuum*)	*C. gloeosporioide*	[52]
	Rs-AFP1 Rs-AFP2	Radish (*R. sativus*)	*Z. bailii*	[53,54]
	IbAMP1 IbAMP 2 IbAMP3 IbAMP4	Touch me not (*I. balsamina*)	*E.coli* *S. enterica * *P. aeruginosa*	[55,56]
	Cp-thionin II	Black-eyed pea (*V. unguiculata*)	*F.* *culmorum* *A. niger* *P. expansum*	[57]
	MsDef1	Alfalfa (*M. sativa*)	*F. graminearum*	[58]
	MtDef4	Barrelclover (*M. truncatula*)	*F. graminearum*	[58]
2S Albumins	Pa-AFP1	Passion fruit (*P. alata*)	*C. gloeosporioides*	[27]
	Pe-AFP1	Passion fruit (*P. edulis*)	*T. harzianum* *A. fumigatus* *F. oxysporum*	[59]
	Pf2	Passion fruit (*P. edulis*)	*F. oxysporum*	[60]
	CW-1	Cheeseweed (*M. parviflora*)	*F. graminearum*	[61]
Glycine-rich proteins	Pg-AMP1	Guava seeds (*P. guajava*)	*Klebsiella* sp. *Proteus* sp. *E. coli*	[62]
Lipid transfer proteins (LTPs)	Ca-LTP1	Chili pepper (*C. annuum* L.)	*C. tropicalis* *F. oxysporum* *C. lindemunthianum* *S. cerevisiae* *P. membranifaciens* *C. tropicalis* *C. albicans*	[63]
	Ha-AP10	Sunflower (*H. annuus*)	*F. solani*	[64]
	Mung bean nsLTP	Black gram (*Phaseolus mungo*)	*F. solani * *F. oxysporum* *S. aureus*	[65,66]
Snakins	Snakin-Z	Jujube fruits (*Z. jujube*)	*S. aureus* *A. niger* *C. albicans* *P. azadirachtae* *P. ultimum* *E. coli* *K. pneumoniae* *B. subtilis*	[67]
	Snakin-1	Potato (*S. tuberosum*)	Antifungal, anti-yeast, and antibacterial	[68,69]
	MsSN1	Alfalfa (*M. sativa*)	*P. fluorescens* *A. tumefaciens* *P. medicaginis* *C. trifolli*	[70]
Thionins	CaThi	Chili pepper (*C. annuum*)	*S. cerevisiae* *C. albicans * *C. tropicalis* *E. coli* *P. aeruginosa*	[71]
	Wheat β-Purothionins	Wheat (*T. aestivum*)	*C. michiganense* *X. campestris*	[72]
	Thionin 2.4	*Arabidopsis * *thaliana*	*F. graminearum*	[73]
	Tu-AMP1 Tu-AMP2	Garden tulip (*T. gesneriana*)	*A. radiobactor* *A. rhizogenes* *C. michiganensis* *C. flaccumfaciens* *F. oxysporum* *G. candidum*	[74]
Cyclotides	Cycloviolacin O2 Cycloviolacin O8	Sweet violet (*V. odorata*)	*X. oryzae* *R. solanacearum* *P. aeruginosa * *F. graminearum*	[75,76,77]
α-Hairpinin-like peptides	LuffinP1	Sponge gourd (*L. cylindrica*)	Translational inhibitory activity	[78]
Hevein-like peptides	Bleogen pB1	Cactus (*Pereskiableo*)	Anti-*Candida*	[79]
	EAFP1 EAFP1	*E. ulmoides*	*P. infestans* *A. lycopersici* *V. dahlia* *G. zeae* *A. nicotianae* *F. moniliforme* *F. oxysporum* *C. gossypii*	[80]
	Ee-CBP	Spindle (*E. europaeus*)	*A. brassicicola* *B. cinerea* *F. culmorum* *N. crassa* *P. exigua*	[81]
	SmAMP3	Chickweed (*S. media*)	*F. solani* *A. alternate* *B. sorokiniana* *B. cinerea*	[82]
Napins	Em2-F18	Jambu fruit (*E. malaccensis*)	*S. aureus* *S. enterica*	[83]
	Tn-AFP1	Water chestnut (*Trapanatans*)	*F. oxysporum*	[84]
	Cn-AMP1	Green coconut (*C. nucifera*)	*M. arachidicola* *P. piricola* *C. tropicalis*	[85]
	Cn-AMP2	Green coconut (*C. nucifera*)	*E. coli* *P. aeruginosa* *S. aureus* *B. subtilis*	[85]
Knottin- type peptides	PAFP-S	Pokeweed (*P. americana*)	*F. oxysporum* *Pyriculariaoryzae* sp.	[86]
	Mj-AMP1 Mj-AMP2	Four o’clock flower (*M. Jalapa*)	*F. oxysporum*	[87]
Unclassified AMPs	Cn-AMP3	Green coconut (*C. nucifera*)	*E. coli* *P. aeruginosa* *S. aureus* *B. subtilis*	[88]

**Table 2:** Application of plant AMPs as food preservatives in the food industry.

Name	Source	Target Organisms	Mechanism of Action	Application in Food Preservation	References
Glycinin basic peptide (GBP)	Soybean	*A. Niger* *Penicillium* sp.	Disruption of the plasma membrane, inhibition of mycelial growth, and spore germination	Fresh wet noodles	[93]
Glycinin basic polypeptides (GBPs)	Soybean	Bacteria	Inhibit the bacterial growth	Japanese Spanish Mackerel fish	[94]
Nine native peptide mixtures	A mixture of pea, lentil, and fava bean flours	*Aspergillus* sp. *Penicillium* sp.	Inhibition of the fungal conidia germination	Wheat bread	[95]
Palm kernel cake peptide mixture	Palm kernel cake	*A. flavus* *A. niger* *Fusarium* sp. *Penicillium* sp.	Disruption of the plasma membrane by increased permeability	Whole wheat bread slices	[96]
Ac-AMP2	*A. caudatus*	*P. expansum*	Inhibition of spore germination	Post harvested pears	[97]
Ala-Tyr peptide	Maize	Bacteria	Inhibition of bacterial growth	Atlantic mackerel fish	[98]
*Momordica charantia* L. seed peptide	*M. charantia*	*S. aureus* *E. coli*	Disruption of bacterial cell membrane	Minced meat products	[99]
Snakin-1	*S. tuberosum*	*Z. bailii*	Disruption of the plasma membrane by increased permeabilization	Fanta orange, cranberry, and apple juice	[69]

**Table 3:** Pros and cons of plant AMPs, in terms of their development for application in the food industry.

	Pros		Cons
1.	Plant AMPs have enormous structural and functional diversity.	1.	The chemical synthesis of AMPs is expensive.
2.	Plant AMPs show diverse mechanisms of action with multiple broad-spectrum bioactivities.	2.	Some plant-derived AMPs show cytotoxicity against different cells.
3.	Hundreds of plant AMPs are already available and enormous scope to find out many more.	3.	Purified plant-derived AMPs show low stability.
4.	AMPs can be easily bioengineered.	4.	Generally, AMPs are immunogenic due to their large size.
5.	Plant AMPs can be used in combination with other food preservatives.	5.	Large-scale production of AMPs is not feasible in the present scenario.

## Data Availability

Not applicable.
